# Probiotics for the improvement of metabolic profiles in patients with metabolic-associated fatty liver disease: A systematic review and meta-analysis of randomized controlled trials

**DOI:** 10.3389/fendo.2022.1014670

**Published:** 2022-11-03

**Authors:** Qiuhong Wang, Ze Wang, Boxian Pang, Huijuan Zheng, Zhengmin Cao, Chunpeng Feng, Wenxin Ma, Junping Wei

**Affiliations:** ^1^ Department of Endocrinology, Guang’anmen Hospital, China Academy of Chinese Medical Sciences, Beijing, China; ^2^ Graduate school, Beijing University of Chinese Medicine, Beijing, China; ^3^ Renal Research Institution of Beijing University of Chinese Medicine, and Key Laboratory of Chinese Internal Medicine of Ministry of Education and Beijing, Dongzhimen Hospital Affiliated to Beijing University of Chinese Medicine, Beijing, China; ^4^ Infections Disease Section, Guang’anmen Hospital, China Academy of Chinese Medicine Sciences, Beijing, China; ^5^ Centre for Evidence-based Chinese Medicine, Beijing University of Chinese Medicine, Beijing, China

**Keywords:** probiotics, metabolic associated fatty liver disease, meta-analysis, randomized controlled trials, liver enzymes, gut microbiota, gut-liver axis

## Abstract

**Objective:**

This meta-analysis of randomized controlled trials (RCTs) was conducted to assess the efficacy of probiotics in the treatment of metabolic-associated fatty liver disease (MAFLD) mainly in terms of liver function, glucose and lipid metabolism, and inflammation.

**Methods:**

RCTs were searched on PubMed, Web of Science, Embase, and the Cochrane Library until June 2022. A meta-analysis was performed on the therapeutic efficacy of probiotics on liver function, glucose and lipid metabolism, and inflammatory biomarkers by using RevMan 5.4 software.

**Results:**

A total of 772 patients from 15 studies were included in the analysis. The methodological quality varied across studies. We found that adding probiotic therapies could reduce the levels of alanine aminotransferase [mean difference (MD): −11.76 (−16.06, −7.46), p < 0.00001], aspartate aminotransferase (MD: −9.08 (−13.60, −4.56), p < 0.0001], γ-glutamyltransferase [MD: −5.67 (−6.80, −4.54), p < 0.00001] and homeostasis model assessment–insulin resistance [MD: −0.62 (−1.08, −0.15), p = 0.01], in patients with MAFLD compared with those in control individuals. However, there was no statistically significant improvement in the levels of total cholesterol, triglycerides, low-density lipoprotein cholesterol, C-reactive protein and tumor necrosis factor α among patients with MAFLD. Subgroup analyses showed that other key factors, such as age, participants’ baseline body mass index, and the duration of intervention, may influence probiotic therapy outcomes.

**Conclusion:**

There is promising evidence that probiotic supplementation can reduce liver enzyme levels and regulate glycometabolism in patients with MAFLD. Further rigorous and long-term trials exploring these novel therapeutic perspectives are warranted to confirm these results.

## Introduction

Non-alcoholic fatty liver disease (NAFLD), characterized by excessive lipid accumulation in the liver without significant alcohol intake or other specific liver damage factors, has become an emerging global health problem. The current global incidence of NAFLD is 20%–33%, and this number is rapidly increasing along with the prevalence of obesity (1–3). NAFLD has been considered to result from metabolic dysfunction (4). Recently, attention has been given to the close correlations between NAFLD and type 2 diabetes mellitus, cardiovascular disease, obstructive sleep apnea, and other metabolic diseases (5). In view of NAFLD being a metabolic disease with extensive multiorgan involvement, metabolic-associated fatty liver disease (MAFLD) has been suggested as a more appropriate overarching term (6). MAFLD may lead to progressive fibrosis, cirrhosis, and hepatocellular carcinoma (7), imposing a heavy burden on individuals, their relatives, and the society overall. Therefore, actively exploring effective strategies for MAFLD prevention and treatment has become a worldwide public health concern.

The pathogenesis of MAFLD is complicated, and its exact mechanism has not been fully elucidated. Its classic pathophysiology is a well-established two-hit hypothesis proposed by Day and James (8) in 1998. According to their hypothesis, the accumulation of triglycerides in liver cells leads to hepatic steatosis, and on this basis, the liver becomes more sensitive to various possible injury factors. This leads to the “second hit”, which leads to inflammation, fibrosis, and cell death in MAFLD. The multiple-hit hypothesis is widely recognized as being more comprehensive and complete in explaining MAFLD pathogenesis than the second-hit hypothesis (9, 10). Among various factors influencing MAFLD pathogenesis, gut microbiota imbalance is drawing increasing research attention. The human gut microbiota is a very large ecosystem, and balanced quantities and species of gut microbiota play an important role in maintaining the intestinal environment. The gut–liver axis refers to the bidirectional connection between the gut and the liver, resulting from the integration of genetic and environmental factors. Their reciprocal communication is established through the portal vein, biliary tract, and systemic circulation (11). Accumulated evidence indicates that gut microbiota alterations can promote intestinal permeability, small intestinal bacterial overgrowth, microbiota-derived mediators, and intestinal dysbiosis (12, 13). Gut–liver axis dysfunctions contribute to the progression and development of MAFLD by mediating processes of inflammation, insulin resistance, bile acids, and choline metabolism (14).

As a result, gut microbiota–targeted therapies have become a new direction in the treatment of MAFLD (15–17). There are various ways to manipulate gut microbiota, among which probiotic supplementation is one of the most highly regarded to improve microbiome homeostasis. The most commonly used probiotics are *Lactobacillus* and *Bifidobacterium*, along with *Lactococcus*, *Streptococcus*, *Enterococcus*, and *Bacillus*. Because multiple clinical trials have shown that probiotic supplementation has definite therapeutic effects on MAFLD, we conducted this meta-analysis of clinical RCT literature to evaluate its efficacy, aiming to provide evidence-based medical information for the clinical treatment of MAFLD.

## Materials and methods

### Database and search strategies

**Figure 1:**
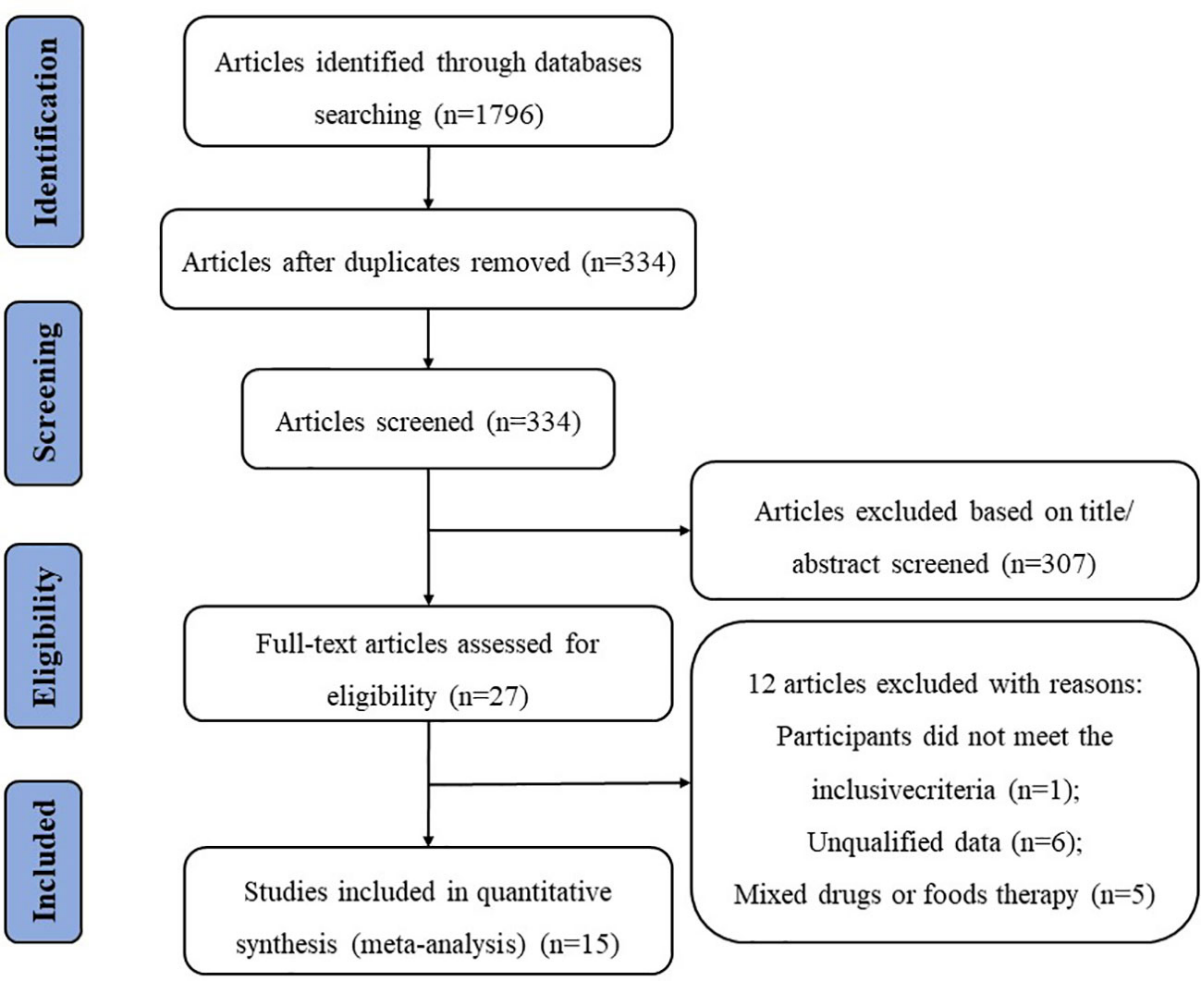
Flow chart of the literature selection process.

We searched for relevant literature published until 30 June 2022, in four electronic databases: PubMed, Web of Science, Embase, and the Cochrane Library. We used the following terms for the database search: (“nonalcoholic fatty liver disease” OR “non-alcoholic fatty liver disease” OR “non-alcoholic steatohepatitis” OR “metabolic associated fatty liver disease” OR “NAFLD”OR “NASH”OR “MAFLD”OR “fatty liver”) AND (“probiotics” OR “probiotic” OR “lactobacillus” OR “bifidobacterium”) AND (“clinical trial” OR “randomized controlled trial” OR “RCT”). The search results were limited to English language publications, and the study selection process is shown in **Figure 1**.

### Inclusion and exclusion criteria

Studies were included according to the following criteria: 1) the design type was randomized controlled trial (RCT); 2) the subjects were patients with NAFLD/non-alcoholic steatohepatitis (NASH) diagnosed by imaging or liver biopsy; and 3) the control group received the same treatment as the experimental group, except for the probiotic preparations. Studies were excluded according to the following criteria: 1) non-RCT; 2) outcomes that had not been clearly stated; 3) the full text or abstract is not available; and 4) reviews, case reports, comments, and conference proceedings.

### Data extraction and literature quality assessment

The electronic literature search and selection were independently conducted by two investigators (B.X.P. and Z.W.). All discrepancies in opinion were resolved by discussion and consensus. When consensus was not reached, a third investigator (Q.H.W) acted as a conciliator. Data of the included literature were extracted (**Table 1**), including the first author, publication year, country, study design, studied population, case number, treatment duration, intervention, and outcomes [including alanine aminotransferase (ALT), aspartate aminotransferase (AST), γ-glutamyltransferase (GGT), total cholesterol (TC), triglyceride (TG), low-density lipoprotein cholesterol (LDL-c), homeostasis model assessment–insulin resistance (HOMA-IR), tumor necrosis factor α (TNF-α), and C-reactive protein (CRP)].

**Table 1:** Characteristics of included randomized controlled trials.

ID	First author, publication year	Country	Study design	Population studied	Case number	Intervention time	Interventions(treatmentgroup)	Interventions(controlgroup)	Outcomes
1	Alisi, 2014	Italy	RCT-DB	Children NAFLD	22/22	16 weeks	*Streptococcus*, *Bifidobacteria*, *Lactobacillus*	Placebo	ALT, HOMA-IR
2	Aller, 2011	Spain	RCT-DB	Adults NAFLD	14/14	12 weeks	*Lactobacillus*, *Streptococcus*	Placebo	HOMA-IR, TC, TG, LDL-c, TNF-α,
3	Behrouz, 2017	Iran	RCT-DB	Adults NAFLD	30/30	12 weeks	*Lactobacillus*, *Bifidobacterium*	Placebo	HOMA-IR
4	Behrouz, 2020	Iran	RCT-DB	Adults NAFLD	30/30	12 weeks	*Lactobacillus*, *Bifidobacterium*	Placebo	ALT, AST, TC, TG, LDL-c, CRP
5	Cai, 2020	China	RCT	Adults NAFLD	70/70	12 weeks	*Lactobacillus*, *Bifidobacterium*, Enterococcus	Non-placebo	ALT, AST, GGT, TC, TG, LDL-c, HOMA-IR
6	Chong, 2021	United Kingdom	RCT-DB	Adults NAFLD	19/16	10 weeks	*Streptococcus*, *Bifidobacterium*, *Lactobacillus*	Placebo	ALT, AST, TC, TG, LDL-c, HOMA-IR, CRP
7	Duseja, 2019	India	RCT-DB	Adults NAFLD	17/13	48 weeks	*Lactobacillus*, *Bifidobacterium*, *Lactobacillus*	Placebo	ALT, AST, TNF-α
8	Famouri, 2017	Iran	RCT-DB	Children NAFLD	32/32	12 weeks	*Lactobacillus*, *Bifidobacterium*	Placebo	ALT, AST, TC, TG, LDL-c
9	Javadi, 2017	Iran	RCT-DB	Adults NAFLD	20/19	12 weeks	*Bifidobacterium*, *Lactobacillus*	Placebo	ALT, AST, GGT
10	Malaguarnera, 2012	Italy	RCT-DB	Adults NASH	34/32	24 weeks	*Bifidobacterium*	Placebo	ALT, AST, HOMA-IR, TC, TG, LDL-c, TNF-α, CRP
11	Manzhalii, 2017	Ukraine	Non-blinded RCT	Adults NASH	38/37	12 weeks	*Lactobacillus*, *Bifidobacterium*, *Streptococcus*	Non-placebo	ALT, AST, GGT, TC, TG
12	Mohamad Nor, 2021	Malaysia	RCT-DB	Adults NAFLD	17/22	6 months	*Lactobacillus*, *Bifidobacterium*	Placebo	ALT, AST, GGT, TC, TG
13	Monem, 2017	Egypt	RCT	Adults NASH	15/15	4 weeks	*Lactobacillus*	Non-placebo	ALT, AST
14	Sepideh, 2015	Iran	RCT-DB	Adults NAFLD	21/21	8 weeks	*Lactobacillus*, *Bifidobacterium*, *Streptococcus*	Placebo	HOMA-IR, TNF-α
15	Vajro, 2011	Italy	RCT-DB	Children NAFLD	10/10	8 weeks	*Lactobacillus*	Placebo	ALT, TNF-α

The methodological quality of the included studies was evaluated by using the Cochrane Collaboration tool. The following six aspects were evaluated: random sequence generation (selection bias), allocation concealment (selection bias), blinding of participants and personnel (performance bias), blinding of outcome assessment (detection bias), incomplete outcome data (attrition bias), and selective reporting (reporting bias). The risk of bias for each item was judged as high, low, or unclear.

### Data and statistical analysis

A meta-analysis was performed using the Review Manager 5.4 software provided by the Cochrane Collaboration. Continuous data were presented as mean difference (MD) with 95% confidence intervals (CIs). The standardized MD (SMD) was used as a summary statistic in this meta-analysis due to the application of different methods to assess the same outcomes. Heterogeneity analysis was performed using the I^2^ statistic. Meta-analysis software (RevManV.5.4) was used, and heterogeneity was evaluated according to I^2^: 25%, 50%, and 75% values were judged as mild, moderate, and substantial heterogeneity, respectively (18). In the absence of significant heterogeneity, data were pooled using a fixed-effects model (I^2^ < 50%); otherwise, a random-effects model was used. Forest plots were used to describe the results, with P-values less than 0.05 considered statistically significant.

## Results

### Characteristics of included studies

The screening process is illustrated in a flow diagram (**Figure 1**). From electronic and manual searches in the four databases, we obtained 1,796 trials, 1,462 of which were duplicates. Among the 334 unduplicated articles, 307 were excluded on the basis of their title or abstract, leaving 27 reports for full manuscript review. Only 15 RCTs (19–33) met our inclusion criteria. The summarized characteristics of the 15 RCTs are shown in **Table 1**. The total number of patients was randomized into probiotic and control (n) groups.

Most of the studies were randomized, parallel-group, and placebo-controlled trials. All clinical trials were double-blind trials except for those conducted by Cai et al. (23), Manzhalii et al. (29), and Monem et al. (31). The 15 trials were published in English between 2011 and 2021, with sample sizes in individual trials ranging from 20 to 140. Most were carried out in Iran (21, 22, 26, 27, 32), with three in Italy (19, 28, 33), one in Spain (20), one in China (23), one in the United Kingdom (24), one in India (25), one in Ukraine (29), one in Malaysia (30), and one in Egypt (31). The participants’ mean ages in the 15 RCTs ranged from 9 to 70 years. Interventions in the included 15 RCTs evaluated different forms of probiotics. A variety of types of bacteria was administered that was mostly based on lactobacilli (14/15), bifidobacteria (12/15), streptococci (5/15), and enterococci (1/15). Probiotics were administered in different forms, including capsules (19, 21, 22, 24–27, 29, 31, 32), powder (23), tablet (20), sachet (28, 30), and unknown (33). The intervention durations also differed among the trials, ranging from 4 to 48 weeks.

### Risk of bias and quality assessment of individual studies

**Figure 2:**
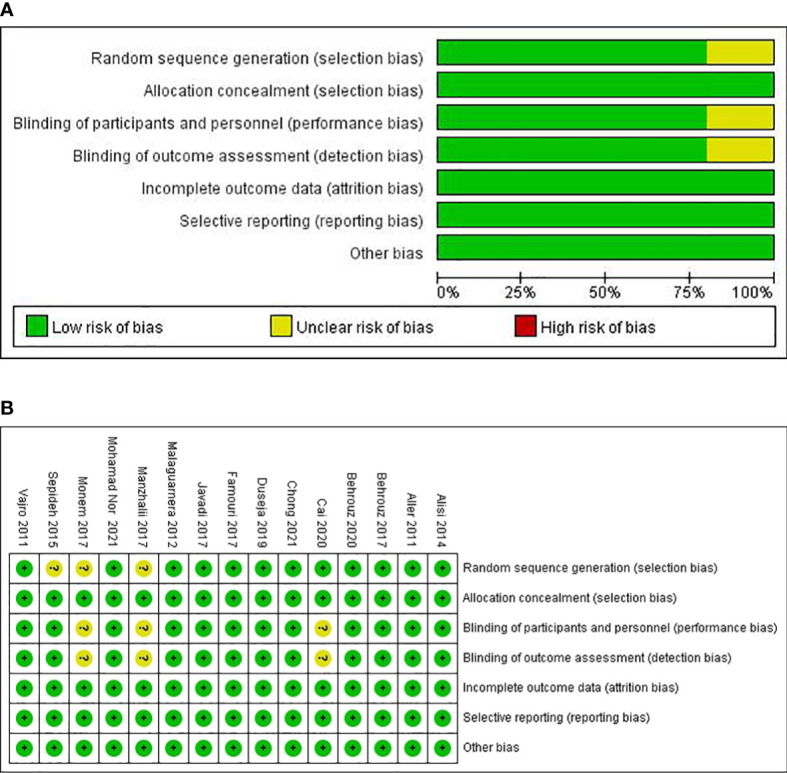
Risk of bias graph **(A)** and risk of bias summary **(B)** for included RCTs.

The risk of bias in the included trials based on different quality domains using the Cochrane Collaboration tool is summarized in **Figure 2**. Among the 15 studies, adequate randomized sequence generation was reported in 80% (12/15) but was unclear in the remaining three (29, 31, 32). The outcome assessors were double-blinded in 80% (12/15) of the trials and were unclear in three trials (23, 29, 31). All the trials had a low risk of bias in allocation concealment. Whereas most of the trials had a low risk of bias in the blinding of participants and key study personnel, three trials (23, 29, 31) had an unclear risk of bias. The outcome assessors were blinded in all of the 15 RCTs. In addition, all the trials showed a low risk of bias based on incomplete outcome data and selective outcome reporting.

### Findings from the meta-analysis

#### Effect on liver function

**Figure 3:**
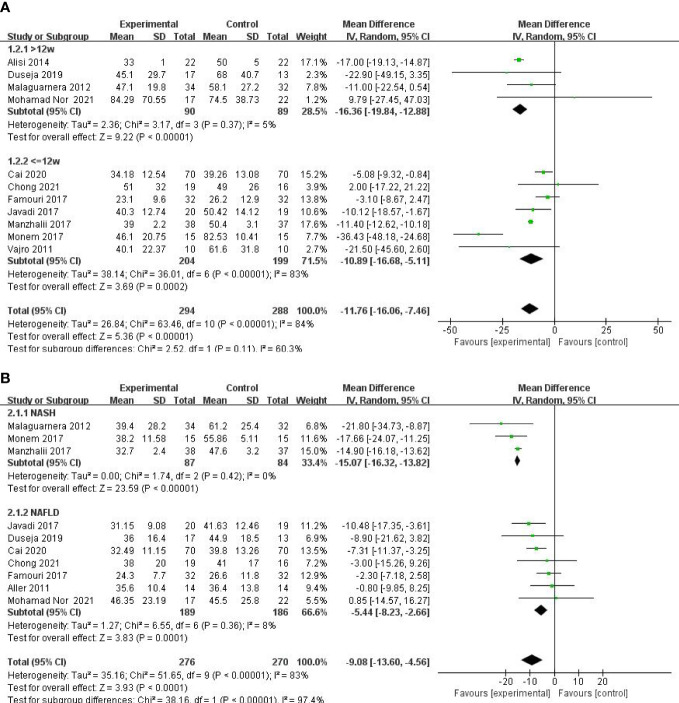
Forest plot of the effect of probiotic supplementation on ALT **(A)**. Forest plot of the effect of probiotic supplementation onAST **(B)**.

A meta-analysis of liver function was performed among studies that reported ALT, AST, and GGT. A total of 11 RCTs involving 582 patients presented the pooled effect of probiotic supplementation on ALT levels. These trials showed heterogeneity in the consistency of the trial results (chi-square = 63.46, p < 0.00001; I^2^ = 84%). Therefore, a random-effects model was used for statistical analysis. Subgroup analysis of the included studies revealed no statistically significant differences between the two groups according to duration (>12 weeks or ≤12 weeks) (P = 0.11). Overall meta-analysis results suggested that probiotic regulation could reduce ALT in patients with MAFLD, as shown in **Figure 3A** [MD: −11.76 (−16.06, −7.46), p < 0.00001]. Sensitivity analysis showed that removing an individual trial did not change the overall effect.

Ten studies including 584 participants presented the pooled effect of probiotic supplementation on AST levels. The trials showed heterogeneity in the consistency of the trial results (chi-square = 51.65, p < 0.00001; I^2^ = 83%). Therefore, a random-effects model was used for statistical analysis. A meta-analysis showed a significant beneficial effect of probiotics compared with the control group in decreasing the level of AST [MD: −9.08 (−13.60, −4.56), p < 0.0001] (**Figure 3B**). On the basis of the type of disease (NASH or NAFLD), our subgroup analysis results showed that the effect of probiotic supplements on AST levels remained significant in studies with NAFLD [−5.44 (−8.23, −2.66), p = 0.0001] and reduced the heterogeneity to 8%. Sensitivity analysis by removing a study (29) showed little change in the outcomes [MD: −8.04 (−11.99, −4.08), p < 0.0001], and heterogeneity was removed (chi-square = 23.23, I^2^ = 66%, p = 0.003).

**Figure 4:**
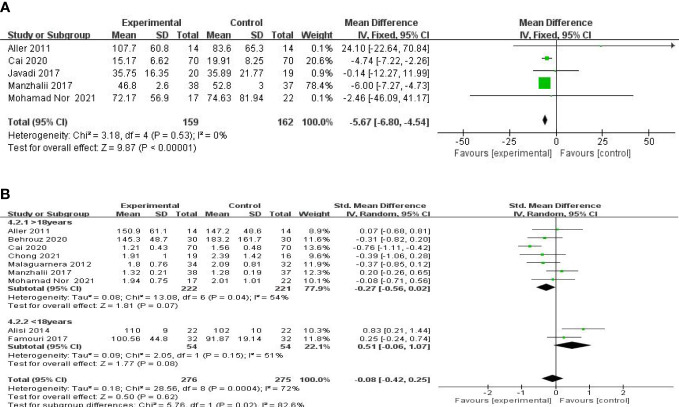
Forest plot of the effect of probiotic supplementation on GGT **(A)**. Forest plot of the effect of probiotic supplementation on TG **(B)**.

Five studies, including 359 participants, presented the pooled effect of probiotic supplementation on GGT levels. The trials showed homogeneity in the consistency of the trial results (chi-square = 3.18, p = 0.53; I^2^ = 0%). Therefore, a fixed-effects model was used for statistical analysis. A meta-analysis showed that, compared with the control group, a significant beneficial effect of probiotic therapies in decreasing the level of GGT [MD: −5.67 (−6.80, −4.54), p < 0.00001] (**Figure 4A**) was detected.

#### Effect on glucose and lipid metabolism

A meta-analysis of liver function was performed among the studies that reported TG, TC, LDL-c, and HOMA-IR. Nine studies, including 606 participants, used the levels of TG to measure outcomes. The trials showed heterogeneity in the consistency of the trial results (chi-square = 28.56, P = 0.0004; I^2^ = 72%). A forest plot showing the results of the meta-analysis on TG is displayed in **Figure 4B**, revealing no significant differences between the experimental and control groups [SMD: −0.08 (−0.42, 0.25), p = 0.61]. Subgroup analysis was carried out as shown in **Figure 4B**. A small decrease in TG levels was found in the studies with mean age over 18 [SMD: −0.27 (−0.56, 0.02), p = 0.07], compared with those with a mean age under 18 [SMD: 0.51 (−0.06, 1.07), p = 0.08].

**Figure 5:**
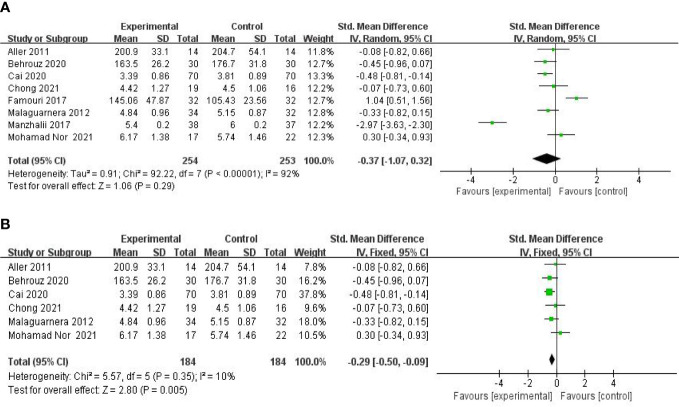
Forest plot of the effect of probiotic supplementation on TC **(A)**. Forest plot of the effect of probiotic supplementation on TC in the included studies with baseline BMIs of more than 27 kg/m2 **(B)**.

Eight studies, including 503 participants, used the levels of TC to measure outcomes. The trials showed heterogeneity in the consistency of the trial results (chi-square = 92.22, p < 0.00001; I^2^ = 92%). Therefore, a random-effects model was used for statistical analysis. The studies showed no significant difference in the reduction of the TC values between the probiotic and control groups [SMD: −0.37 (−1.07, 0.32), p = 0.29] (**Figure 5A**). However, the body mass index (BMI) seemed to be an important factor. Further subgroup analysis of the BMI showed a significant beneficial effect of synbiotic supplementation on TC levels for BMIs of more than 27 kg/m^2^ [SMD: −0.29 (−0.50, −0.09), p = 0.005] (**Figure 5B**). Findings from sensitivity analysis revealed that excluding an individual study did not change the overall effect.

**Figure 6:**
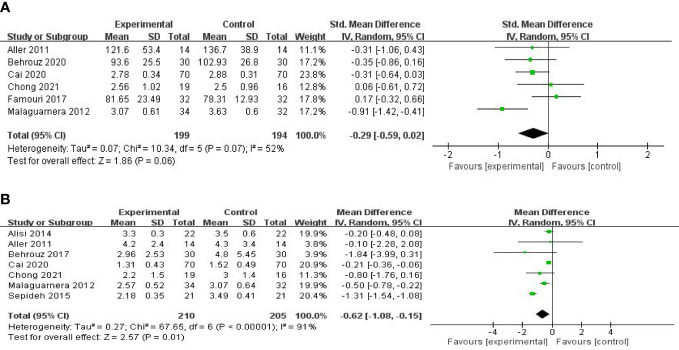
Forest plot of the effect of probiotic supplementation on LDL-C **(A)**. Forest plot of the effect of probiotic supplementation on HOMA-IR **(B)**.

Six studies, including 393 participants, used the levels of LDL-C to measure outcomes. The trials showed heterogeneity in the consistency of the trial results (chi-square = 10.34, p = 0.07; I^2^ = 52%). Therefore, a random-effects model was used for statistical analysis. There were no significant differences in the reduction of LDL-C values between the probiotic and control groups [SMD: −0.29 (−0.59, 0.02), p = 0.06] (**Figure 6A**). Sensitivity analysis by removing a study (26) altered the overall estimates [SMD: −0.39 (−0.61, −0.17), p = 0.0005].

**Figure 7:**
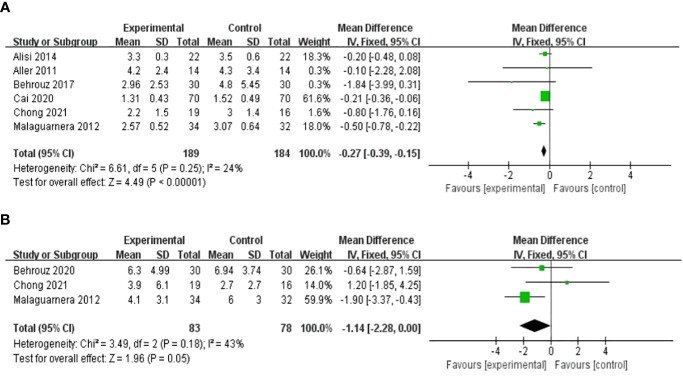
Forest plot of the effect of probiotic supplementation on HOMA-IR in studies with a duration of more than 8 weeks **(A)**. Forest plot of the effect of probiotic supplementation on CRP **(B)**.

Seven studies, including 415 participants, used the levels of HOMA-IR to measure outcomes. The trials showed heterogeneity in the consistency of the trial results (chi-square = 67.65, p < 0.00001; I^2^ = 91%). Therefore, a random-effects model was used for statistical analysis. The use of probiotics could significantly reduce the levels of HOMA-IR [MD: −0.62 (−1.08, −0.15), p = 0.01] (**Figure 6B**). When the meta-analysis was subgrouped by the duration of the study (>8 weeks or ≤8 weeks), our subgroup analysis results showed that the effect of probiotic supplements on HOMA-IR levels remained significant in studies with a duration of more than 8 weeks [MD: −0.27 (−0.39, −0.15), p < 0.00001] (**Figure 7A**), and heterogeneity was removed (chi-square = 6.61, I^2^ = 24%, p = 0.25). Sensitivity analysis by removing one study (32) altered the overall estimates [MD: −0.27 (−0.39, −0.15), p < 0.00001].

#### Effect on biomarker of inflammation

Three RCTs involving 161 patients presented the pooled effect of probiotic supplementation on the CRP level. These trials showed homogeneity in the consistency of the trial results (chi-square = 3.49, p = 0.18; I^2^ = 43%). Therefore, a fixed-effects model was used for statistical analysis. There were no significant differences in the reduction of CRP values between the probiotic and control groups [MD: -1.14 (-2.28, 0.00), p = 0.05] (**Figure 7B**).

**Figure 8:**
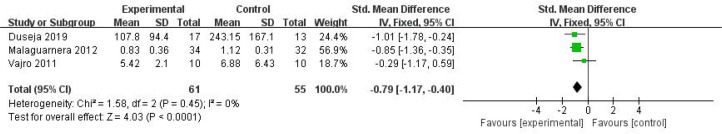
Forest plot of the effect of probiotic supplementation on TNF-α in the included studies with baseline BMIs of less 28 kg/m2.

Five studies, including 186 participants, used TNF-α levels to measure outcomes. The trials showed heterogeneity in the consistency of the trial results (chi-square = 27.83, P < 0.0001; I^2^ = 86%). Therefore, a random-effects model was used for statistical analysis. The use of probiotics could slightly reduce TNF-α levels [SMD: −0.78 (−1.64, 0.07), p = 0.07]. Subgroup analysis was carried out on the basis of the participants’ baseline BMI. A significant decrease in TNF-α levels was found in studies with baseline BMIs of less 28 kg/m^2^, as shown in **Figure 8** [SMD: −0.79 (−1.17, −0.40), p < 0.0001].

### Publication bias

The number of trials included was too small to conduct any sufficient additional analysis of publication bias.

## Discussion

NAFLD has attracted considerable attention in the realm of endocrine and metabolic diseases. As there are no targeted drugs at this stage, NAFLD treatment primarily relies on lifestyle modification. Therefore, actively exploring the effective strategies for the prevention and treatment of NAFLD has become a worldwide public health concern. Because the occurrence and development of NAFLD are highly related to metabolic disorders, some experts have suggested using the term MAFLD instead of NAFLD (6, 34). The “multiple-hit” pathological hypothesis has been widely recognized in recent years (9, 10). Some studies (35–37) have shown that abnormalities in the gut–liver axis, including intestinal microecology imbalance, intestinal bacterial overgrowth, intestinal permeability increase, or intestinal leakage, play a role in the occurrence and development of NAFLD. Probiotic supplementation for patients with NAFLD is aimed at restoring the normal gut microbiota, thereby reducing liver inflammation. This may be the rationale for treating NAFLD using probiotics (38, 39).

This study systematically reviewed and quantitatively summarized scientific evidence on the use of probiotic treatments for NAFLD. A total of 772 patients from 15 different clinical studies were included in the review, in which trials involving adult and pediatric patients were analyzed and reported separately to assess the effects of probiotic supplements on liver enzymes, the glucose and lipid metabolism index, and systemic inflammatory biomarkers in patients with NAFLD. A comprehensive analysis indicated that probiotic supplementation could decrease the level of liver enzymes and the glycometabolic index (AST, ALT, GGT, and HOMA-IR) compared with those in the placebo group. However, probiotic supplementation seems to have no significant influence on the lipid index (TC, TG, and LDL-c) and proinflammatory biomarkers (CRP and TNF-α).

In this review, we found that probiotics were closely associated with a decrease in AST, ALT, and GGT levels, suggesting that probiotic supplementation in NAFLD patients may have protective effects on liver function by regulating the composition and metabolism of the gut microbiota. This conclusion is consistent with a previous meta-analysis by Loman et al. (40), which involved 11 trials of probiotic intervention in patients with MAFLD and found that probiotic intervention resulted in significant reductions in ALT, AST, and GGT levels. ALT, AST, and GGT are markers of liver injury, not markers of a specific liver function. Therefore, the mechanism leading to the reduction of their concentration in microbial therapy may be multifactorial. Although there is substantial evidence for the efficacy of the microbial treatment of liver diseases in animals and humans, the mechanism for a probiotic-mediated reduction in serum liver enzymes has not been fully elucidated (41, 42).

Regarding the effects of probiotic supplementation on glucose metabolism, we found that probiotics could reduce the levels of HOMA-IR in patients with MAFLD compared with those in the control groups. However, there is a high degree of heterogeneity in HOMA-IR. This may be a result of differences between race, age, gender, duration of intervention, BMI, and complicated diseases. For instance, one study (43) suggested that non-Hispanic Whites and African Americans displayed greater insulin sensitivity than East Asians and South Asians. Moreover, women were found to be intrinsically more insulin-resistant than men in another study, possibly due to specific sex-linked genes and differences in metabolic control elements (44). As for the intervention of probiotic supplementation on the lipid profiles of patients with MAFLD, our findings are consistent with those of Xiao et al. and Sharpton et al. (45, 46), in which probiotic supplementation did not improve some indicators of lipid profiles in patients with MAFLD. By contrast, in the study by Tang et al. (47), LDL-c levels were significantly decreased after probiotic intervention. We noticed that the levels of TG did not significantly differ between patients with MAFLD and control individuals, regardless of the random-effects model and sensitivity analysis used to minimize heterogeneity. These differences may be formed as a result of clinical heterogeneity, including study protocol, dosage form, and characteristics of the research population.

In our study, probiotic treatment had no significant effect on CRP and TNF-α. These are important indicators of the protective effect of probiotics on patients with MAFLD (48–50) The results of our study are not consistent with those of Pan (39), Khan (51), Gao (52), and Huang (53). In a meta-analysis, probiotic supplementation did not improve some measures of inflammation in patients with MAFLD (54, 55). It is worth noting that the individual differences in the subjects may lead to different outcomes along with a diversity of probiotic interventions, including dosage, duration of intervention, drug formulation, and treatment combinations used in different studies. Some studies (56–58) have shown that the gut microbiota of older adults has more interindividual variation and that the composition differs compared with that of younger adults. For instance, age-related changes in the gut microbiota were found as a lower Firmicutes-to-Bacteroidetes (F:B) ratio in older adults as compared with younger adults, along with a reduction in species producing SCFAs(short-chain fatty acids). Differences in gut microbiota composition, functional genes, and metabolic activities were also observed between obese and lean individuals (59). There is evidence suggesting that individuals with obesity have a greater *Lactobacillus*, proteobacteria, firmicutes, and F:B ratio and less verrucomicrobia, faecalibacterium, bacteroidetes, *Methanobrevibacter smithii*, *Lactobacillus plantarum*, and paracasei (60).

Previous meta-analysis studies (47, 61) have shown similar results, reporting the benefits of probiotics in improving hepatic steatosis, liver enzymes, lipid profiles, plasma glucose, HOMA-IR, cytokines, and the extent of hepatic fatty infiltration in patients with MAFLD, which could be considered as a promising therapeutic approach. Our meta-analysis showed significant improvements in various parameters after probiotic treatment in different RCTs. However, the magnitude of improvement was not consistent among these studies because of the use of different strains, dosing patterns, and intervention duration. Subgroup analyses were also performed on the basis of probiotic strain and treatment time to address the unexplained high heterogeneity between studies. According to research findings, long-term intervention may have a good effect on improving liver enzyme indexes, glucose and lipid metabolism indexes, and inflammatory indexes. Consensus is urgently needed in terms of the type, dosage, and duration of probiotics treatments. It is known that inconsistencies in baseline characteristics (including age, BMI, and disease severity) between studies that could influence the gut microbiome may explain the high degree of variation in treatment response for each outcome variable. In general, although the underlying therapeutic mechanisms are not completely clear, probiotics remain a promising option for the treatment of MAFLD. This meta-analysis provided supporting evidence for the use of probiotics in the clinical treatment of MAFLD in the future.

### Strengths and limitations of this study

The efficacy of probiotics in reversing gut–liver axis dysfunction by regulating gut microbiota has generated increasing interest in probiotic treatment for patients with MAFLD. In conclusion, this study conducted a systematic review and meta-analysis of the literature on probiotics treating patients with MAFLD to evaluate the effect of probiotics on liver function, glucose and lipid metabolism, and inflammation. MAFLD is nearly an equivalent concept to NAFLD to some extent. Both NAFLD and its subtypes (NASH) treated by probiotics were all included in this study. We performed subgroup meta-analyses and assessments of the effects of treatment duration, baseline BMI of subjects, and age on the overall effect size. However, the sample size of some literature included in this study was small, and some data, such as quartiles, were difficult to merge, which led to an incomplete analysis. The heterogeneity among various studies may be due to intraindividual strain differences, treatment time, optimal dosage of probiotics, treatment type, use of prebiotics, individual genotypes, and other factors.

## Conclusion

Our meta-analysis showed that probiotic consumption among patients with MAFLD has a beneficial effect on the metabolic indicators by significantly reducing the levels of ALT, AST, GGT and HOMA-IR; however, this intervention had no statistically significant effect on the levels of TG, TC, LDL-c, CRP and TNF-α. Furthermore, more rigorous and larger RCTs are needed to specify the strains of probiotics, as well as the changes in gut microbial composition, to determine the efficacy of probiotic supplementation in the control of liver function, glucose and lipid metabolism, and inflammation in patients with MAFLD.

## Data availability statement

The original contributions presented in the study are included in the article/supplementary material. Further inquiries can be directed to the corresponding authors.

## Author contributions

QW and JW designed the study. BP and ZW searched databases and performed the selection of studies. QW, ZW and HZ analyzed the data and wrote the manuscript. ZW, BP and WM critically evaluated the review and commented on it. QW, CF, ZC and WM contributed to the revised version. All authors contributed to the article and approved the submitted version.

## Funding

This study was supported by Young Elite Scientists Sponsorship Program by CAST (Grant No. 2019QNRC001) and Special project for training outstanding young scientific and technological talents of the Chinese Academy of Chinese Medical Sciences (Grant No. ZZ14-YA-010) and The Scientific and Technological innovation project of China Academy of Chinese Medical Sciences (Grant No.CI2021A01612), but this study did not receive any public funding from commercial institutions.

## Conflict of interest

The authors declare that they have no conflicts of interest and no financial interests related to the material in this manuscript.

## Publisher's note

All claims expressed in this article are solely those of the authors and do not necessarily represent those of their affiliated organizations, or those of the publisher, the editors and the reviewers. Any product that may be evaluated in this article, or claim that may be made by its manufacturer, is not guaranteed or endorsed by the publisher.
